# Discovery of Novel and Differentially Expressed MicroRNAs between Fetal and Adult Backfat in Cattle

**DOI:** 10.1371/journal.pone.0090244

**Published:** 2014-02-28

**Authors:** Jiajie Sun, Yang Zhou, Hanfang Cai, Xianyong Lan, Chuzhao Lei, Xin Zhao, Chunlei Zhang, Hong Chen

**Affiliations:** 1 College of Animal Science and Technology, Northwest A&F University, Shaanxi Key Laboratory of Molecular Biology for Agriculture, Yangling, Shaanxi, China; 2 Institute of Cellular and Molecular Biology, Jiangsu Normal University, Xuzhou Jiangsu, China; University of Queensland, Australia

## Abstract

The posttranscriptional gene regulation mediated by microRNAs (miRNAs) plays an important role in various species. Recently, a large number of miRNAs and their expression patterns have been identified. However, to date, limited miRNAs have been reported to modulate adipogenesis and lipid deposition in beef cattle. Total RNAs from Chinese Qinchuan bovine backfat at fetal and adult stages were used to construct small RNA libraries for Illumina next-generation sequencing. A total of 13,915,411 clean reads were obtained from a fetal library and 14,244,946 clean reads from an adult library. In total, 475 known and 36 novel miRNA candidates from backfat were identified. The nucleotide bias, base editing, and family of the known miRNAs were also analyzed. Based on stem-loop qPCR, 15 specific miRNAs were detected, and the results showed that bta-miRNAn25 and miRNAn26 were highly expressed in backfat tissue, suggesting these small RNAs play a role in the development and maintenance of bovine subcutaneous fat tissue. Putative targets for miRNAn25 and miRNAn26 were predicted, and the 61 most significant target transcripts were related to lipid and fatty acid metabolism. Of interest, the canonical pathway and gene networks analyses revealed that PPARα/RXRα activation and LXR/RXR activation were important components of the gene interaction hierarchy results. In the present study, we explored the backfat miRNAome differences between cattle of different developmental stages, expanding the expression repertoire of bovine miRNAs that could contribute to further studies on the fat development of cattle. Predication of target genes analysis of miRNA25 and miRNA26 also showed potential gene networks that affect lipid and fatty acid metabolism. These results may help in the design of new intervention strategies to improve beef quality.

## Introduction

MicroRNAs (miRNAs) are a class of small regulatory noncoding RNAs with an average length of 22 nucleotides, which were first identified in *C. elegans* [1] and were reported to act as endogenous regulators of protein-coding genes at the posttranscriptional level in a sequence-specific manner [2]. MiRNAs have been implicated in fundamental biological processes such as differentiation, proliferation, apoptosis, and homeostasis [3]. In addition, recent reports have also suggested that miRNAs play crucial roles in the regulation of adipogenesis. In 2003, one report suggesting that miR-14 was involved in fat metabolism. The deletion of *mir-14* results in animals with increased levels of triacylglycerol and diacylglycerol, whereas increases in the number of *mir-14* copies have the converse effect [4]. Another study demonstrated that miR-143 is involved in adipocyte differentiation and may act by targeting the expression of gene *extracellular-regulated protein kinase 5* (*ERK5*) [5]. Furthermore, the miR-8/miR-200 transcripts promote adipogenesis by inhibiting Wnt signaling, which is a negative regulator of adipogenesis [6], [7]. In addition to miR-122, miR-370, miR-378/378*, miR-27, and miR-335 have also been recently associated with lipid metabolism and adipocyte differentiation [8]–[12]. MiR-122 inhibition in high-fat fed mice was found to result in reduced plasma cholesterol levels, which was linked to a reduction in cholesterol synthesis and the stimulation of fatty-acid oxidation [8]. MiR-370 has similar effects as miR-122 on lipid metabolism by targeting *carnitine palmitoyl transferase* (*Cpt1a*) to reduce fatty acid oxidation [9]. The overexpression of miR-378/378* during adipogenesis increases the accumulation of triacylglycerol [10]. By contrast, miR-27 inhibits the expression of peroxisome proliferator-activated receptor (PPAR) γ and C/EBPα, the two master regulators of adipogenesis [11]. In contrast, increased miR-335 expression has been associated with an elevated body, liver and white adipose tissue weight, and hepatic triglyceride and cholesterol [12]. Later studies have also demonstrated miR-33′s role in the regulation of lipid metabolism, while inhibition of miR-33 resulted in increased fatty acid oxidation and the reduced accumulation of fat stores [13], [14]. However, the major role of miRNAs in regulating lipid metabolism and adipogenesis remains unknown.

Release 19 of mirBASE (http://www.mirbase.org) contains 21,264 entries representing hairpin precursor miRNAs, expressing 25,141 mature miRNA products in 193 species. However, the number of known bovine miRNAs is limited, with only 755 reported compared with 2,042 from humans and 1,281 from mice. Cattle are an important species in the animal production industry. They have tremendous importance not only for food production, but also as a mammalian model organism for comparative genomics and biological studies [15]. Additionally, lipid deposition, especially in subcutaneous adipose depots, is directly associated with yield grade and meat quality [16]. Previous studies have provided limited insight into the miRNA population present in bovine species by only investigating general characteristics, expression patterns, and features of their target genes [17]–[21]. In 2010, Jin *et al.* reported that approximately 20% of the miRNAs involved in adipogenesis and lipid deposition were identified as being correlated with backfat thickness. Their results suggest that miRNAs play a regulatory role in white adipose tissue development in beef [22].

Given the emerging roles of miRNAs in fat tissue development in multiple species, identifying the differentially expressed miRNAs is an important first step to investigating the function of miRNAs in the course of bovine lipid metabolism and adipogenesis. In the present study, using both next-generation sequencing and reverse transcription, quantitative PCR (RT-qPCR) assays were employed to characterize the genome-wide miRNA expression profile in bovine backfat between fetal and adult periods. We sought to identify a panel of fat depot-specific miRNAs that could serve as targets for further study with a long-term goal of using this information to control backfat deposition in fed cattle.

## Results

### Deep sequencing of bovine short RNAs

**Figure 1:**
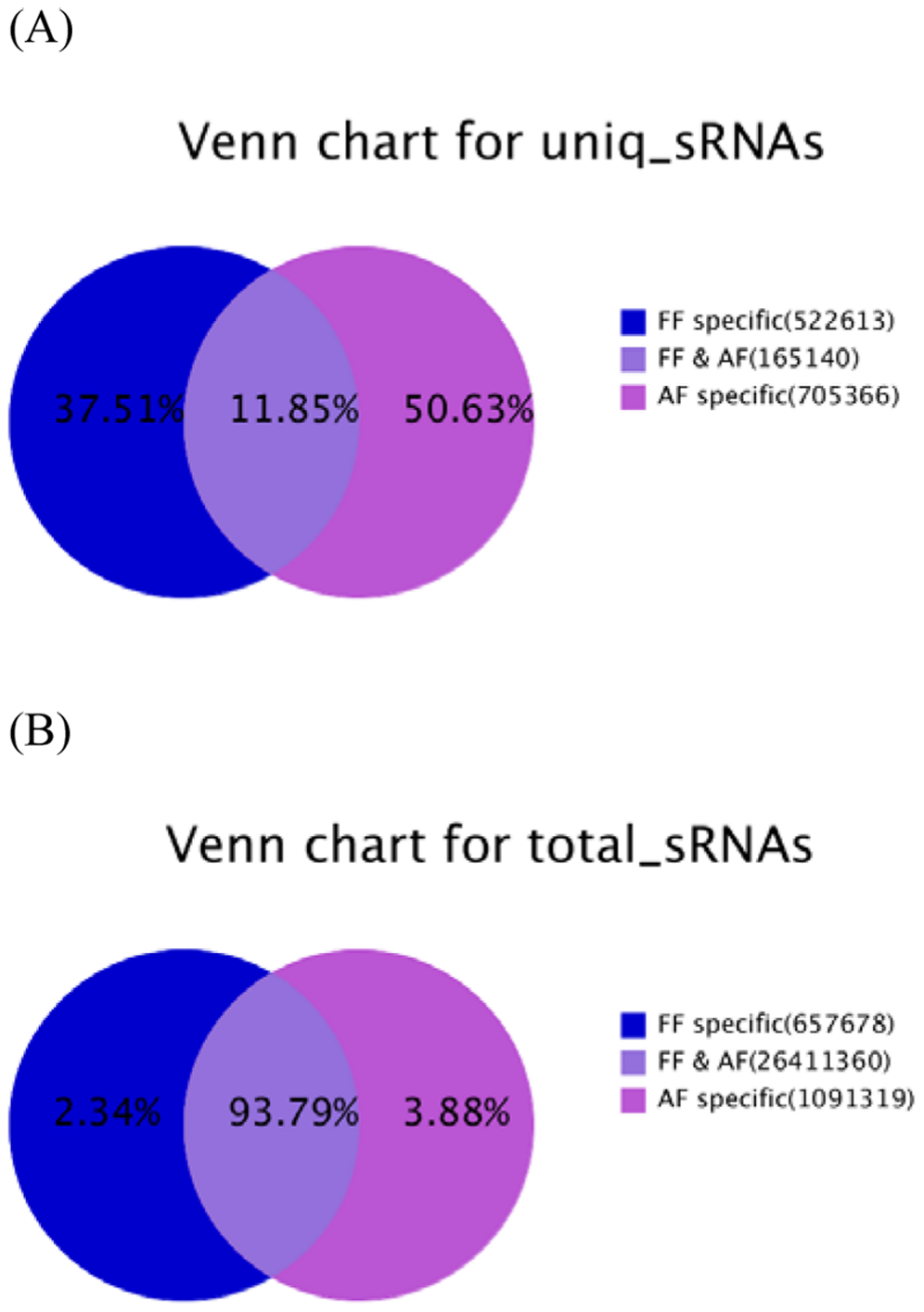
Length distribution of small RNAs in the fetal bovine (gray) and adult bovine (black) libraries.

**Figure 2:**
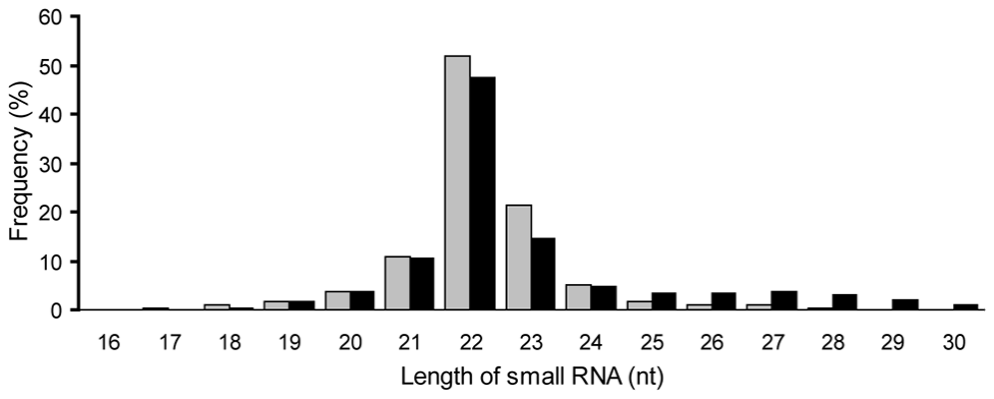
Summary of the common and specific tags of two samples, including the summary of unique tags (A) and total tags (B).

In order to identify novel and differentially expressed miRNAs in the bovine backfat at fetal and adult stages, two small RNA libraries were constructed for Solexa SBS technology sequencing. Solexa sequencing provided a total of 14,071,065 and 14,373,930 reads of 3 nt–30 nt from the fetal and adult backfat tissue libraries, respectively. After removing the low quality reads, adaptors, and insufficient tags and sequences, a total of 13,915,411and 14,244,946 clean reads of 18–30 nt were obtained (Table 1). The unique and total numbers of the common and tissue-specific small RNA sequences in the two libraries are shown in Figure 1. The fetal-specific unique sequences account for 37.51% of all sequence reads and 50.63% in the adult library, respectively (Figure 1A). The percentages of the fetal-specific and adult-specific sequences were 2.34% and 3.88% of the total number of small RNAs in the two libraries (Figure 1B). Length distribution analysis showed that most reads ranged from 21 to 24 nt, which is typical of the small RNA of Dicer-processed product. The size distribution (18 nt–30 nt) of the small RNA from the fetal and adult stages in Chinese Qinchuan cattle was similar (Figure 2). For example, in the fetal period, the 22 nt and 23 nt sequences were the dominant small RNAs, which accounted for 51.80% and 21.46% of the total sequences as well as 47.42% and 14.68% in the adult bovine library.

**Table 1:** Summary of small RNA sequencing date.

Type	Fetal bovine backfat		Adult bovine backfat	
	Count	%	Count	%
Total_read	14071065		14373930	
High_quality	14004677	100%	14303707	100%
Adaptor3_null	8126	0.06%	7508	0.05%
Insert_null	1780	0.01%	2165	0.02%
Adaptor5_contaminants	25722	0.18%	20528	0.14%
Small_than_18 nt	53582	0.38%	28490	0.20%
PolyA	56	0.00%	70	0.00%
Clean_reads	13915411	99.36%	14244946	99.59%

Next, all of the reads were aligned against the *Bos taurus* genome (Btau_6.0) using the SOAP Program [23]. A total of 9,063,361 reads were matched to the bovine genome in the fetal library (127,542 unique sRNAs) and a total of 9,690,034 reads were in the adult library (250,668 unique sRNAs). Subsequently, the genome-matched small RNA sequences were clustered into several RNA classes such as known miRNAs, degraded fragments of mRNA, repeats, rRNA, tRNA, snRNA/snoRNA, and others (Table 2). Known bovine miRNAs accounted for 59.17% of all sequence reads in the fetal library and 47.89% in the adult library, suggesting that mature miRNAs were highly enriched in our small RNA libraries. However, after analyzing the number of unique sequences, the proportion of small RNA sequences derived from known miRNAs represented only a very small fraction of the total number of unique transcripts (0.56% and 0.44% in the fetal and adult libraries). The highest fraction of unique sequences (70.89% and 64.46% in the two libraries) was unclassified small RNA sequences, which probably included novel miRNA candidates and other classes of regulatory RNAs.

**Table 2:** Distribution of the genome-mapped sequence reads in small RNA libraries.

Locus class	Fetal bovine backfat				Adult bovine backfat			
	Unique sRNA	Percent (%)	Total sRNA	Percent (%)	Unique sRNA	Percent (%)	Total sRNA	Percent (%)
Total	687753	100%	13915411	100%	870506	100%	14244946	100%
miRNA	357	0.05%	496	0%	385	0.04%	683	0%
Exon_antisense	23294	3.39%	29572	0.21%	67318	7.73%	94168	0.66%
Exon_sense	6521	0.95%	8108	0.06%	12650	1.45%	14366	0.10%
Intron_antisense	12719	1.85%	19684	0.14%	28458	3.27%	37886	0.27%
Intron_sense	3827	0.56%	8234182	59.17%	3857	0.44%	6821393	47.89%
rRNA	116439	16.93%	768966	5.53%	136492	15.68%	2630720	18.47%
repeat	12587	1.83%	26041	0.19%	28962	3.33%	78032	0.55%
scRNA	325	0.05%	10067	0.07%	517	0.06%	17019	0.12%
snRNA	3838	0.56%	9407	0.07%	6382	0.73%	24007	0.17%
snoRNA	1932	0.28%	10112	0.07%	3009	0.35%	13710	0.10%
srpRNA	964	0.14%	5291	0.04%	1666	0.19%	21352	0.15%
tRNA	17413	2.53%	105611	0.76%	19699	2.26%	175326	1.23%
Unknown	487537	70.89%	4687874	33.69%	561111	64.46%	4316284	30.30%

### Known bovine miRNAs expressed in backfat

Currently, miRBase 19.0 lists 766 miRNA precursors and 755 mature miRNAs sequences cloned or predicted in *bos taurus* (http://www.mirbase.org). To identify conserved miRNAs in our dataset, all small RNA sequences with a length of 18–30 nucleotides were Blastn searched against the known bovine mature miRNAs and their precursors in the miRNA database miRBase. In sum, 3,890 unique sequences (8,234,368 reads) were annotated as miRNA candidates in the fetal bovine library as well as 3,926 unique sequences (6,821,671 reads) in the adult bovine library, while the rest were unannotated. The miRNA candidates were then clustered into 432 and 412 categories corresponding to 457 and 442 independent genomic loci in the two libraries according to sequence similarity (Table 3), of which 369 miRNAs overlapped in both libraries (Table S1). Each category included multiple homologs, which differed in sequence length by only 1–5 nucleotides. Such homologous sequences with different lengths are thought to be variants produced by biochemical modifications and the imprecise processing of either primary or precursor miRNAs by Drosha and Dicer enzymes. In addition, the most abundant miRNA family was let-7, accounting for about 86% and 82% of the total sequence reads from the fetal and adult libraries, respectively.

**Table 3:** Summary of known miRNA in each sample.

	miR	miR-5p	miR-3p	pre-miRs	Unique matched to pre-miRs	Read matched to pre-miRs
Known miRs	598	79	78	766		
Fetal library	336	46	50	457	3890	8234368
Adult library	328	40	44	442	3926	6821671

Analyses of the first nucleotide bias of the 18–30 nt miRNA candidates revealed that uridine (U) was the most common first nucleotide of the known 18 nt (91.42%), 25 nt (99.83%), and 27 nt (100%) miRNAs, whereas adenine (A) was the most common one of the known 19 nt (91.90%) and 22 nt (98.49%) miRNAs in the fetal stage. In the adult stage, U was the dominant first nucleotide of the 18 nt (100%), 19 nt (98.35%), and 25 nt (99.85%) miRNAs, while A was the dominant first nucleotide of the 22 nt (96.67%) and 26 nt (100%) nucleotide miRNAs (Figure S2). Additionally nucleotide bias analysis at each position showed a high frequency of G+C content at the 2nd, 4th, 5th, 8th, 11th, 15th and 20th positions, at 97.00%, 95.22%, 94.85%, 93.89%, 94.12%, 97.19%, and 94.45% respectively, while nucleotides A+U were distributed mainly in the remaining positions with the exception of the 12th, 19th and 23th positions in the fetal stage. Equally studies have shown similar results in the adult bovine stage (Figure S2).

Position 2∼8 of a mature miRNA is called the seed region, which is highly conserved. The target of miRNA might be different with the change of nucleotides in this region [24]. In our analysis pipeline, miRNAs that might have base edit can be detected by aligning unannotated sRNA tags with mature miRNAs from miRBase19, allowing one mismatch at a certain position. The results show that approximately 40.28% in the fetal bovine library and 42.23% in the adult bovine library of the identified miRNA sequences were found to have mismatches that might have been caused by post-transcriptional modification and/or RT-PCR as well as sequencing errors (Table S2 and S3). Obviously, highly abundant miRNAs (bta-let-7a/b/c/e/f, bta-miR-154c and bta-miR-199a-3p) had been higher edited probability in backfat tissue. This indicated that highly-expressed miRNAs targeted more genes in this tissue. Accordingly, nucleotide changes in the seed region could increase miRNA diversity, change their targets, and may have important phenotypic consequences. In addition, many variants were observed from the 5′ or 3′ ends of the annotated miRNA sequences, which were apparently generated from the same precursor. Most end variants differed by one or several nucleotides at the 3′ end nucleotide, and a small percentage of them differed at the 5′ end (Table S4). Generally, the sequence reads of variants are less abundant than those of annotated miRNA sequences. However, in some cases, the variants are more abundant, indicating that they are probably dominant and might substitute for the reported miRNA sequences in the miRBase. For example, the annotated let-7e sequence was 21 nt long and had 17,378 reads in our libraries, whereas its most abundant 22 nt variant had 64,998 reads. Similarly, miR-21, miR-103, miR-107, miR140, miR-143, miR-152, miR-199, miR-432, miR-839, and miR-2284x were predominantly found at lengths of 22, 21, 21, 23, 21, 21, 21, 21, 22, and 22 nt, respectively.

We investigated the chromosome locations (BTAU6.0) for 475 known miRNAs in the fetal and/or adult libraries. Most of the conserved miRNAs were found on the autosomes or X chromosome of the cattle. However, 16 and 15 miRNAs from the respective fetal and adult bovine backfat tissues failed to match the genomic sequences, which might be due to an incomplete assembly model of the bovine genome. In detail, the shortest chromosome, 25, and the longest chromosome, 1, encode 12 and 14 miRNAs, respectively, corresponding to 0.28 and 0.08 miRNAs per 1 Mbp genomic sequences. Chromosome 21 has the greatest number of known miRNAs distributed in the cattle genome (69 miRNAs). The X chromosome has the second greatest number of the known miRNAs, accounting for 8.00% (38/475) of the total miRNAs. It is noteworthy that the expressed miRNAs in the chromosomes X and 21 are dominant in the two libraries (Table S1). To date, 25,141 mature miRNA products have been identified in 193 species. In our study, further analysis identified a total of 432 and 412 conserved miRNAs that belonged to 236 miRNA families in fetal and adult bovine libraries. The identified miRNA families have been shown to be conserved in a variety of species. For example, let-7, miR-1, miR-25, miR-9, and miR-25 families have been found in 68, 69, 68, 72, and 68 species, respectively, while miR-2363, miR-3432, miR-3604, miR-6526, and miR-6536 families have only been detected in *bos taurus* (Table S5). The largest miRNA family size identified was miR-2284, which consisted of 27 members; miR-2285, let-7, miR-30, and miR-376 possessed 22, 8, 6, and 5 members, respectively, whereas other miRNA families such as miR-107, miR-122, miR-140, and miR-1839 had only one member (Table S1). In addition, different family members also displayed drastically different expression levels. For example, the abundance of the miR-2284 family varied from 0 (bta-miR-2284e) to 57,585 reads (bta-miR-2284x) with deep sequencing in the fetal stage. The existence of a dominant member in an miRNA family may suggest that the regulatory role of this family was performed by the dominant member at the developmental time when the samples were collected for RNA extraction. Abundance comparisons of different members in an miRNA family may provide valuable information on miRNAs' role in that specific stage of bovine development.

**Figure 3:**
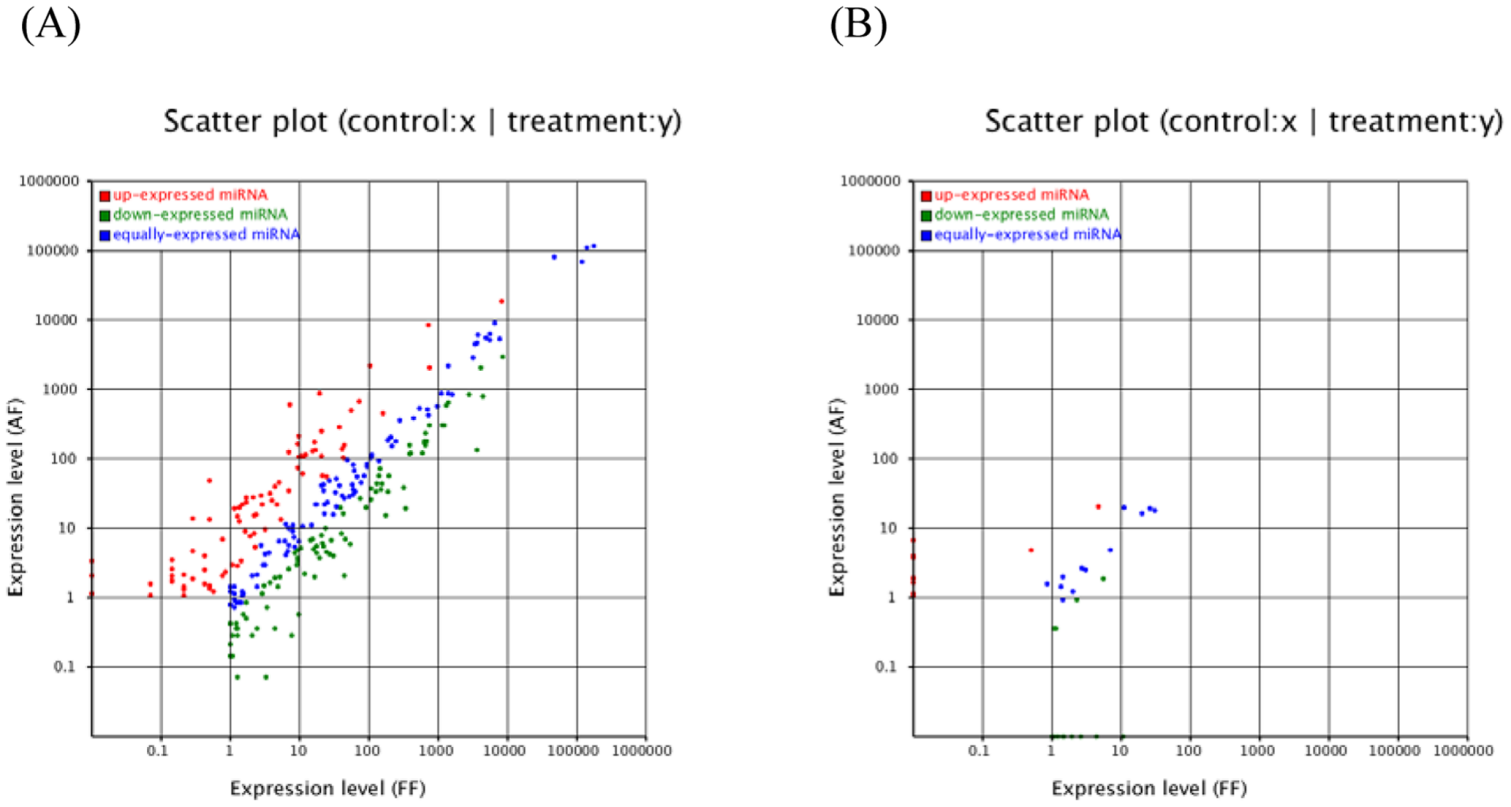
The differentital expressions of bovine conserved (A) and novel (B) miRNAs between fetal and adult bovine backfat tissue were shown. Note: Expression level (FF): Expression level of fetal bovine; Expression level (AF): Expression level of adult bovine. Each point in the figure represents a miRNA. Red points represent miRNAs with fold change >2, blue points represent miRNAs with 1/2< fold change ≤2, and green points represent miRNAs with fold change ≤1/2.

The expression of known miRNAs in the two samples was demonstrated by plotting the log2-ratio and scatter plots (Figure 3A). The expression profiles between the two libraries were shown in Table S6. According to the results, 173 miRNAs consisted of 87 up-expressed miRNAs and 86 down-expressed miRNAs. These were significantly different between the two libraries. For example, the expression of miR-101, miR-432, and miR-185 was higher in fetal bovine backfat tissue than the patterns shown by miR-199a, miR-503, and miR-154c in adult bovine backfat tissue. This suggests that these miRNAs may affect the growth and development of fat tissue.

### Newly identified miRNAs in backfat

The miRNA hairpins are mostly located in intergenic regions, introns, or in the reverse repeat sequence of the coding sequence. The characteristic hairpin structure of miRNA precursor can be used to predict novel miRNA. We used the prediction software Mireap to predict novel miRNA by exploring the secondary structure, the Dicer cleavage site, and the minimum free energy of the unannotated small RNA tags that could be mapped to genome. Mireap can be accessed from the following link: http://sourceforge.net/projects/mireap/. Seven of the key conditions for predicting novel miRNA are as follows [25]: (1) the tags that be used to predict novel miRNA are the unannotated tags that can match to reference genome, the tags that can align to the intron region, and the tags that can align to the antisense exon region; (2) hairpin miRNAs can fold secondary structures, and mature miRNAs are present in one arm of the hairpin precursors, which will be considered as candidate miRNA genes; (3) the mature miRNA strand and its complementary strand (miRNA*) present 2 nt 3′ overhangs; (4) hairpin precursors lack large internal loops or bulges; (5) hairpins' secondary structures are steady, with a free energy of hybridization lower than or equal to −18 kcal/mol; (6) miRNA precursors with secondary structures have higher minimal free energy indexes (MFEIs) than other different types of RNAs. Based on Solexa sequencing, we identified 36 novel bovine miRNAs that corresponded to 63 genomic loci. Ten novel miRNAs were in the fetal bovine library and nine were in the adult bovine library; 17 of these overlapped in both libraries (Table S7). An examination of pre-miRNAs and other RNAs (tRNA, rRNA, and mRNA) revealed that miRNAs were significantly different from other RNAs [26]. Specifically, most miRNA precursors had an MFEI greater than 0.85, significantly higher than tRNAs (0.64), rRNAs (0.59), or mRNAs (0.65). The results suggested that the MFEI can easily be used to distinguish miRNA from other non-coding and coding RNAs. This provides a more precise criterion to predict miRNAs using computational approaches, and in our database approximately 41 pre-miRNAs had an MFEI greater than 0.85. In addition, the expression profiles of the novel miRNA genes we obtained by measuring the sequencing frequencies are shown in Figure 3B, indicating that 24 miRNAs consisted of 10 up-expressed and 14 down-expressed miRNAs.

### miRNAs differentially expressed between adult bovine muscle and backfat

In an earlier miRNA discovery study, we observed 521 miRNAs including 104 novel miRNA candidates from the Chinese Qinchuan bovine longissimus thoracis [21]. In order to identify the fat-specific miRNAs involved in bovine lipid metabolism and adipogenesis, we compared the backfat library with the muscle library and found that miRNA expression was different in the two tissues. In the muscle, bta-let-7a/b/f, bta-miR-1, and bta- miR-206 were the dominant expressed miRNAs, with more than 100,000 reads. They constituted 96.83% of the total known miRNA sequencing reads, suggesting that they have abundant in muscle tissue. The sequencing frequencies of the 58 miRNAs (*e.g.*, bta-miR-122, bta-miR-1185, bta-miR-1224) were much lower (1≤ sequencing reads ≤10). However, in fat tissue, seven miRNAs (bta-let-7a/b/c/e/f, bta-miR-154c, and bta-miR-199a-3p), each with more than 100,000 reads, were the most abundant. In the two libraries, a total of 233 known miRNAs were co-expressed. Among the known miRNAs, only one fat-specific and 17 muscle-specific miRNAs were found (Table S8A), and the expression levels of the tissue-specific expressed miRNAs were low. In comparison with miRNA expression in muscle tissue, 195 miRNAs in backfat tissue were significantly up-regulated, while 28 were significantly down-regulated (Figure S2A). In addition, five novel miRNAs were identified, and the results are shown in Table S8B. The expression levels of novel miRNAs in the muscle and backfat tissue were also shown in Table S8C and Figure S2B. The read number for each novel miRNA was much lower than that of the majority of conserved miRNAs. For instance, bta-miRn26 and bta-miRn11 were both novel miRNAs, and the total reads were only 286 and 15 in the backfat library, respectively. However, the total reads of two conserved miRNAs (bta-let-7a and bta-miR-154c) were striking, at 1,636,370 and 120,128, respectively. This might suggest that these novel miRNAs play a specific role during the developmental stages. Since this study investigated Chinese Qinchuan bovine muscle and backfat tissue miRNAs, whether these low-abundant miRNAs are expressed at higher levels in other tissues and organs remains to be investigated.

### Validation of miRNA expression

**Figure 4:**
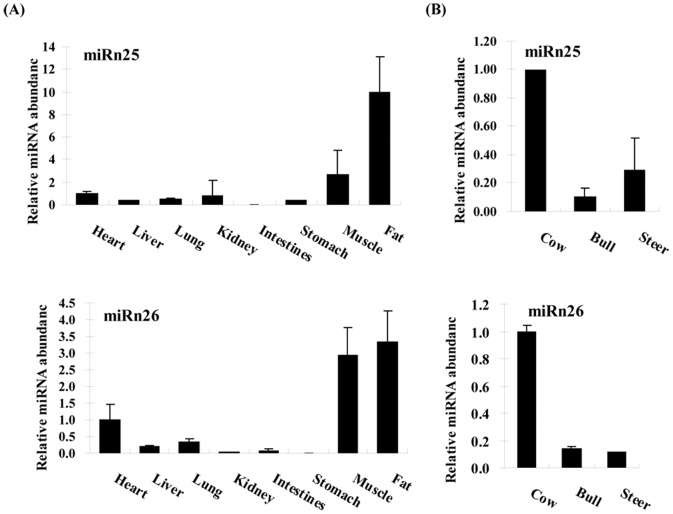
The expression of miRNAs in bovine different tissues (A) and different sex (B).

To validate our sequencing data, stem-loop qPCR [26] analysis of miRNA expression was performed in the heart, liver, lung, kidney, intestines, stomach, muscle, and backfat of female animals. At least five animals were used for each sample. The expression levels of the 15 miRNAs were determined, and the selected miRNAs included five reported tissue-specific miRNAs (bta-miR-9, miR-124, miR-122, miR-27, and miR-103), seven backfat-predominant miRNAs in fetal and/or adult bovine backfat libraries (let-7a, miR-140, miR-199a, miR-320, miR-2284x, miRn8, and miRn25) as well as three high-read miRNAs (miR-154, miR-1839, and miRn26) in backfat that were compared with the muscle library. A comparison of miRNA expression profiles among tissues revealed that very few miRNAs had tissue-specific expression (*e.g.*, miR-122 and -2284x in the liver). Several miRNAs were found to be highly expressed in particular tissues: miR-1839 in the heart, miR-103 in the liver, miR-9 and -154 in the kidneys, miRNAn25 in fat, and miRNAn26 in muscle and fat tissue, respectively. In contrast, another seven miRNAs were found in six or more tissues, and the two most abundant miRNAs across the eight tissues were miRNA-199a and miRNA-320 (Figure 4A and Figure S3). To further explore the highly expressed miRNAs between cattle of different genders, we also performed a quantitative analysis of the miRNAs in adult cow, bull, and steer backfat. miRNAn25 and miRNAn26 were expressed at significantly higher levels in cow than bull and steer backfat (Figure 4B).

### Prediction of target genes

The miRNA expression patterns in different tissues have been investigated. In cattle, miR-9 and miR-124 in the brain, miR-122 in the liver, and miR-1, miR-133a, and miR-206 in muscle are all tissue-specific [27]. In the present study, miRNAn25 and n26 were also specifically identified in backfat. The similar expression pattern indicates that tissue-specific miRNAs may have certain roles in the respective tissues. We therefore could postulate that miRn25 and n26 may influence lipid metabolism and adipogenesis in backfat tissue. To identify the potential function of the two novel miRNAs, target prediction was performed using RNAhybrid software. A total of 2,416 putative target sites for miRNAn25 (Table S9A) and 672 target sites for miRNAn26 (Table S10A) were identified in the cattle's backfat, respectively. Next, all the target gene candidates were submitted for homology and annotation searches and Gene Ontology (GO) annotation using an online version of the Blast2GO program (www.Blast2GO.com). In total, 2,416 target sites of miRNAn25 were annotated with 1,818 GO terms (Table S9B), and 841 GO terms (Table S10B) were associated to 672 target sites of miRNAn26 in the Non-Redundant database. The distributions of GO term categories for the target genes of miRNAn25 and miRNAn26 were highly similar (Figure S4). The top five biological functions identified by the Blast2GO program included categories related to a wide variety of physiological and biological events, such as cellular processes, metabolic processes, biological regulation, responses to stimulus, and multicellular organismal processes. Further analyses revealed that 126 miRNAn25 target genes relating to lipid metabolism were annotated with 32 GO terms (Table S9C), and 44 target sites for miRNAn26 were annotated within 10 GO terms (Table S10C), respectively.

**Figure 5:**
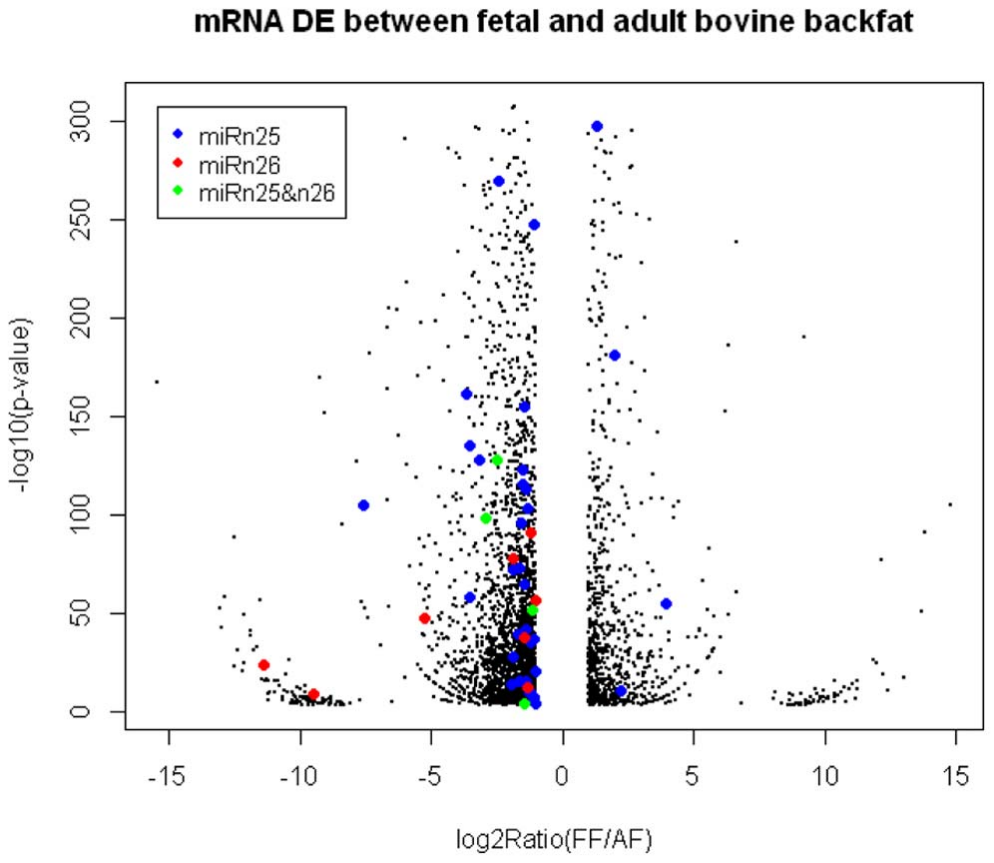
Gene expression plot between fetal and adult bovine backfat tissue were shown. Note: Each dots in the figure represents a gene. Green dots represent the target genes of bta-miRNAn25, red dots represent bta-miRNAn26, and blue dots represent bta-miRNAn45&48, respectively.

Previous reports have documented the functional connection between RNA editing and miRNA-mediated post-transcriptional gene silencing [28]. Therefore, to characterize the potential effects of RNA editing on miRNAs, we first identified differentially expressed transcripts between fetal and adult bovine backfat based on RNA-Seq technology. In total, 7,495,616 reads could be uniquely aligned in the fetal library and 6,088,485 reads were in the adult library, representing ∼18.04 Gbp and ∼14.89 Gbp of expressed sequences in the respective libraries. Of the 3,879 known genes that were expressed in bovine backfat, 62 and 95 were differentially expressed at fetal and adult stages, respectively. Of those, 26% up-regulated and 74% down-regulated transcripts were identified by comparing the adult stage to the fetal stage (Table S11). Additionally, 40.47% miRNAn25 target genes related to lipid metabolism were expressed in the fetal and/or adult stages (Table S9D), as well as 36.36% target genes for miRNAn26 (Table S10D), respectively. An MA-plot indicated that most of the target genes related to lipid and fatty acid metabolism were down-regulated, accounting for about 91.80% of the total transcripts (Figure 5).

**Figure 6:**
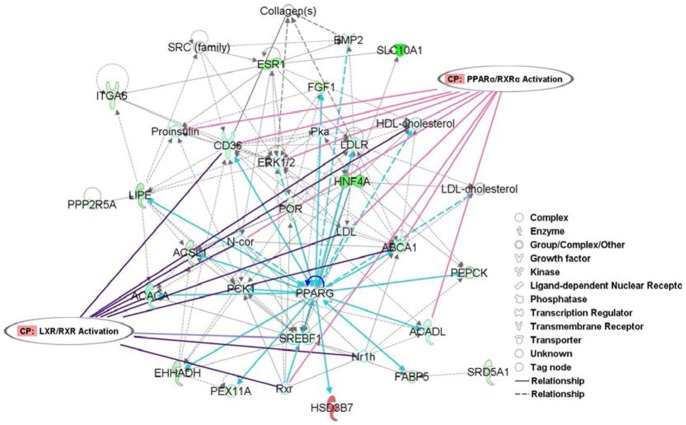
Functional and pathways analysis using miRNAn25&n26 target genes related to lipid and fatty acid metabolism as input date. The node colour indicated the expression of genes: (red) up-regulated and (green) down-regulated in adult stage relative to fetal stage. The shapes of nodes indicated the functional classes of the gene products. Relevant canonical pathways that feature modulated genes were indicted as well (*e.g.* LXR/RXR Activation and PPARα/RXRα Activation).

The 61 most significantly different miRNAn25 and n26 target transcripts related to lipid and fatty acid metabolism were investigated using Ingenuity Pathways Analysis software (IPA, www.ingenuity.com), and the results are shown in Figure 6. In comparison with the adult stage, five of these transcripts had increased gene expression in the fetal stage and 56 had decreased expression. Briefly, genes that play a crucial role in lipid synthesis (*ABCA1*, *ACACA*, *ACADL*, *BMP2*, *ESR1*, *FGF1*, and *SRD5A1*), lipid accumulation (*CD36*, *LDLR*, and *SFEBF1*), lipid homeostasis (*EHHADH* and *HNF4A*), fatty acid metabolism (*PPARG*, *ACSL1*, *SLC10A1*, *FOX* and *LDLR*), fatty acid oxidation (*LIPE* and *FOR*), and lipid efflux (*SREBF1*) were down-regulated in the fetal stage rather than in the adult stage. On the contrary, the gene involved in fatty acid concentration, lipid uptake, and steroid synthesis (*HSD3B7*) was up-regulated. When comparing the fetal stage with the adult stage, the most representative canonical pathways significantly modulated in bovine backfat were involved in peroxisome proliferator-activated receptor α/retinoid X receptor alpha (PPARα/RXRα) and farnesoid X receptor/retinoid X receptor (FXR/RXR) activation, etc. (Table 4).

**Table 4:** Description of the top ten canonical pathways significantly modulated in bovine backfat tissue when comparing fetal stage with adult stage.

Ingenuity canonical pathways	Genes
PPARα/RXRα Activation	ACADL, CD36, ABCA1, ERK1/2, HDL-cholesterol, LDL, N-cor, Pka, Proinsulin, Rxr
FXR/RXR Activation	HDL-cholesterol, PPARG, SLC10A1, SREBF1, HNF4A, LDL, Nr1h, PEPCK, Proinsulin, Rxr
LXR/RXR Activation	LDLR, SREBF1, CD36, ACACA, ABCA1, HDL-cholesterol, LDL, N-cor, Nr1h, Rxr
Hepatic Cholestasis	HDL-cholesterol, SLC10A1, SREBF1, HNF4A, ESR1, HSD3B7, N-cor, Pka, Proinsulin, Rxr
LPS/IL-1 Mediated Inhibition of RXR Function	SLC10A1, FABP5, SREBF1, ALAS1, ACSL1, ABCA1, HDL-cholesterol, LDL, Nr1h, Rxr
RAR Activation	RDH10, PIK3R1, BMP2, ERK1/2, HDL-cholesterol, LDL, N-cor, Rxr, SRC
TR/RAR Activation	LDLR, HDL-cholesterol, SREBF1, N-cor, PEPCK, ACACA, Rxr, PCK1
AMPK Signaling	PIK3R1, LIPE, ACACA, PPP2R5A, ERK1/2, Pka, SRC, Proinsulin
ERK/MAPK Signaling	ERK1/2, ESR1, Pka, PPARG, PPP2R5A, SRC
PPAR Signaling	ERK1/2, N-cor, Nr1h, PPARG, Proinsulin, Rxr

Note: Statistical significance of pathway modulation was calculated via a right-tailed Fisher's Exact test in Ingenuity Pathway analysis and represented as –log (P value): -log values exceeding 1.30 were significant FDR<0.05.

## Discussion

Adipose tissue is a highly specialized structure in energy storage. In mammals, the adipose tissue pool is composed of at least two functionally different types of fat: white and brown fat. White adipose tissue is the primary site of energy storage and of the release of hormones and cytokines [29]. Excess accumulation of white adipose tissue causes obesity. Brown adipose tissue, on the other hand, affects whole-body metabolism and may alter insulin sensitivity [30] and modify susceptibility to weight gain [31]. However, brown adipose tissue is primarily only found in fetuses and young individuals and is believed to have no physiologic relevance in adults, resulting in significant differences between fetal and adult adipose tissue development. In addition, potential regulators of adipogenesis include miRNAs, which encode an abundant class of ∼22 nucleotide evolutionarily conserved RNAs that control gene expression at the posttranscriptional level by targeting mRNAs for degradation and/or translational repression [32]. Computational and experimental analyses suggest that miRNAs may regulate the expression of about 30% of human and mouse genes [33]. Recently, miRNA expression profiles and functions have been extensively investigated, but little is still known about the role of miRNAs in metabolic tissues, particularly adipose tissue. Of particular relevance, miR-14 [34] and miR-278 [4] in the fat of flies regulate lipid metabolism, and miR-122 in the liver of mice controls triglyceride metabolism and cholesterol biosynthesis [8]. In the current investigation, we therefore hypothesized that miRNA expression would precede de novo lipogenesis in adipose tissue. To address our hypothesis, fetal and adult Chinese Qinchuan bovine backfat samples were collected, and two miRNA libraries were constructed for Solexa SBS technology sequencing.

Of the mappable sequences, the majority of the small RNAs were 21–24 nt in size, which coincided with the known specificity for Dicer processing and the features of mature miRNAs [35]. In the present study, the 22 nt sequences in bovine backfat (including the fetal and adult samples) were the dominant small RNAs. This was in agreement with Chen *et al.*, who reported that 21–23 nt sequences was significantly greater than others, and almost half of the sequences in the backfat of Large White and Meishan pigs were canonical 22 nt miRNA [36]. Recently, similar results in Qinchuan bovine [21], Texel [37], and carp [38] muscle and goat mammary glands [39] have also been reported. However, our findings are not consistent with the previous studies on bovine testis and ovaries [40], [41] and even of those on maize [42]. The differences observed between our study and previous studies may have been due to different experimental approaches (Solexa Sequencing vs random cloning), different species (cattle vs maize), and different tissues (adipose tissue vs testis and ovary tissues). In addition, the variations in sequence length may be also attributed to differences in functional roles, such as RNA editing in miRNA-mediated gene silencing [43], 3′-editing [44], and degradation of microRNAs by a family of exoribonucleases [45].

The XX/XY chromosomes are a biological system that determines the development of sexual characteristics in most mammals. We calculated the densities of the miRNAs on the chromosomes using the miRNA data from the miRBase and found that there were no available data on the Y chromosome in cattle. This was supported by a previous deep-sequencing study in cattle [40] and sheep [37]. The bovine X chromosome is 149 Mbp in size, ranking the 2nd largest among all of the chromosomes. In this study, 38 unique miRNAs were located on the X chromosome, accounting for 8% (38/475) of genome-mapped miRNAs. The density distribution of miRNA loci on the X chromosome reached 0.25 miRNA loci per Mbp, which ranked 3rd in the density distribution of miRNA loci among all of the chromosomes. Higher densities of miRNAs on X chromosomes were also identified in eight other mammalian species [46], consistent with prior studies that demonstrated their resistance to meiotic sex chromosome inactivation [47].

MiRNAs from adipose tissue of distinct breeds of cattle had different levels of expression and backfat thickness [22]. Further, a recent study showed variations in miRNA profiles among different locations of subcutaneous backfat, and in beef cattle, miR-9 and miR-124 in the brain and miR-122 in the liver were all tissue-specific [27]. In the present study, these miRNAs were also identified, and the results suggest that some of the previously reported tissue-specific miRNAs maybe have certain limits in terms of their distribution in tissues. Liver-specific miR-122 has a diversity of roles in liver, *e.g.*, metabolism, hepatocarcinogenesis [48], cholesterol, and fatty-acid metabolism in the adult liver [8]. Consistent with previous results, miR-122 had the most abundant expression level in the liver (Figure S3). Although physiological roles of most miRNAs are largely unknown, recent evidence indicates that they are also associated with the regulation of insulin sensitivity for the treatment of type 2 diabetes and obesity [49], fat accumulation [50], and adipocyte differentiation [51], [52]. For example, miR-103 is a negative regulator of insulin sensitivity by targeting caveolin-1, which is a critical regulator of the insulin receptor [49]. Over-expressed miR-27 allows activated hepatic stellate cells to restore their ability to accumulate cytoplasmic lipid droplets [50], and miR-27 expression has been found to block the expression of PPARγ and C/EBPa, the two master regulators of adipogenesis [51]. These data strongly suggest that miR-27 represents a new class of adipogenic inhibitors and may play a role in the pathological development of obesity. In addition, let-7 regulates adipocyte differentiation in part by targeting the transcription factor high-mobility group AT-hook 2 (HMGA2), thereby promoting the transition of preadipocytes from clonal expansion to terminal differentiation [52]. In this study, we also focused on these three miRNAs related to lipid and fatty acid metabolism, and their expression levels were determined in eight different tissues by stem-loop qPCR. The results show that they were not significantly expressed in bovine backfat (Figure S3). Such an expression pattern indicates that they may have more dramatic functions, *e.g.*, miR-27 in signaling and immune responses [53], miR-103 in the regulation of neuronal migration by modulating *Cyclin-dependent kinase 5, regulatory subunit 1* (*CDK5R1*) expression [54], and let-7 in developmental timing in *Caenorhabditis elegans* [55]. Based on the Solexa sequencing results, six high-read miRNAs between animals of different stages were selected from the bovine backfat libraries for further study: miR-140 and -2284x were notably higher in the fetal stage, and miR-199a, -320, n8, and n25 were higher in the adult stage. Additionally, miR-154, -1839, and n26 were also retained, which were specifically expressed in the adult bovine backfat library rather than the muscle library. Using q-PCR assay, we found some miRNAs influenced mainly by tissue location, such as miR-2284x, which had liver-specific expression, while miRn25 and n26 were highly expressed in backfat (Figure 4A and Figure S3). The tissue specificity of these miRNAs is likely related to the regulatory peculiarities of each tissue. miRNAs tissue specificity has recently been reported in other species including mice [56], [57] and humans [58], indicating the need for different regulatory mechanisms to address the unique physiology of each tissue type.

Bulls have less fat than cows and steers, resulting in less tasty meat, particularly because bulls are slaughtered at a younger physiological age than other meat-producing cattle. In addition, they have higher levels of energy expenditure and protein retention than cows and steers, contributing to a lower availability of nutrients for fat deposition. Nowadays, molecular biology techniques, especially genomics, continue to further expand our knowledge of the adipocyte. In addition, new genetic markers, transcription factors, and genes are continuously being discovered [59]. In order to further expound the key mechanisms that can act to improve fat level between cattle of different sexes, highly expressed miRn25 and n26 in bovine backfat were analyzed in adult cow, bull, and steer backfat tissues, and that they were expressed at significantly higher levels in cow tissue (Figure 4B). The expression patterns and level of miRn25 and n26 in the tested bovine tissues suggest that these miRNA may be more relevant to the highly conserved biological process in different sexes. Further research is needed to uncover their regulatory functions. Interestingly, most of the potential novel miRNAs discovered in this study were expressed at low levels (Table S8C), explaining why they were not discovered in previous efforts and showing the advantage of next-generation sequencing in miRome analysis. This pattern of expression is consistent with the previous results of human studies [60].

To better understand the functions of the identified miRNAs in lipid metabolism and adipogenesis, putative targets of the highly expressed miRn25 and n26 in bovine backfat were predicted (Table S9A and S10A). According to the analysis and annotation, targets of miRn25 and n26 had diverse functions, ranging from genes encoding transcription factors involved in signal transduction, enzymes involved in metabolism, various kinases, and isomerase and helicase to genes regulating oxidative reduction and transport (Table S9B and S10B). Further analysis identified that 61 most significantly different miRNAn25 and n26 target transcripts related to lipid and fatty acid metabolism overlapped with the bovine backfat transcriptome profile obtained by RNA-Seq (Table S9D and S10D). More specifically, an important target was the PPARγ gene (Figure 6), which was a dominant regulator of adipogenesis and fat cell gene expression [61]. Other important genes targeted by the highly expressed miRNAs included *acyl-CoA synthetase long-chain family member 1* (*ACSL1*), *lipase, hormone-sensitive* (*LIPE*), *CD36 molecule (thrombospondin receptor)* (*CD36*), and *acetyl-CoA carboxylase alpha* (*ACACA*), which are all known to be involved in lipid metabolism and adipogenesis. We therefore hypothesized that miRNAn25 and n26 can target sequences in these genes to regulate the development of bovine fat tissue.

Finally, the identified target genes were mapped to the genetic networks, and an IPA network score of 49 and 23 genes of interest (GOI) presented functions related to lipid metabolism, small molecule biochemistry and molecular transport (Figure 6). Many of these GOI have been previously associated with lipid metabolism and adipogenesis (e.g. fatty acid oxidation and lipid accumulation and homeostasis). These results support the utility of our methods for identifying the significant genes related to this biological function. Additionally, several GOI related to lipid and fatty acid metabolism that appear to be novel in the context of the IPA network were also identified, providing new targets for future study. These genes included *Collagen(s)*, *ERK1/2*, *HDL-cholesterol*, *LDL*, *LDL- cholesterol*, *N-cor*, *Nr1h*, *PEPCK, Pka*, *Proinsulin*, *Rxr*, and *SRC*. We will take our analysis one step further and determine which genes were likely the most critical in the bovine backfat. Two well-characterized signaling pathways stood out in our gene interaction hierarchy (GIH): PPARα/RXRα activation and LXR/RXR activation. PPARα controls fatty acid degradation [62], and sterol regulatory element-binding protein-1c activated by liver X receptor (LXR) regulates fatty acid synthesis [63]. Therefore, our GIH can also provide a context for evaluating the potential of current mechanisms involved in bovine fat development.

## Conclusions

We have identified 475 known miRNAs and 36 novel miRNAs in the backfat of fetal and adult Qinchuan bovines using deep sequencing technologies. This study expands the repertoire of bovine miRNAs and could initiate further study in the fat development of cattle. In addition, the miRNA expression patterns among eight tissues in beef cattle showed that most miRNAs are ubiquitously expressed, suggesting that these miRNAs may play a role in a broad range of biological processes in various tissues. However, miRn25 and n26 were significantly expressed in bovine backfat, suggesting that they are likely to play a role in the development of bovine fat tissue and could be potential molecular markers for genetics and breeding. Putative targets for miRn25 and n26 were predicted, and the 61 most significantly different target transcripts related to lipid and fatty acid metabolism were identified using the Blast2GO program. Canonical pathways and gene network analysis revealed that PPARα/RXRα activation and LXR/RXR activation stood out in our gene interaction hierarchy. The results obtained may help in the design of new selection strategies to improve beef quality.

## Materials and Methods

### Ethics statement

All animal protocols were approved by Institutional Animal Care and Use Committee (IACUC) of Northwest A&F University and Qinbao Animal Husbandry Co., Ltd, respectively. All surgery was performed under sodium pentobarbital anesthesia, and all efforts were made to minimize suffering. Bovine embryos of slaughtered cows were collected from a local slaughterhouse of Xi'An, P.R. China. The adult Qinchuan bovine tissues were obtained from Qinbao Animal Husbandry Co., Ltd, which is a cattle breeding and slaughtering corporation in Xi'An, P.R. China.

### Tissue collection and high-throughput sequencing

On day 200 (d200), bovine embryos (gestation period 280 days) were collected into sterile physiological saline immediately after removal from the reproductive tract of slaughtered cows at a local abattoir. Fetal age was estimated based on crown-rump length [64]. Bovine tissue samples including the heart, liver, lungs, kidneys, stomach, intestine, muscle, and fat were collected from the adult Chinese Qinchuan cattle. These tissues were snap-frozen in liquid nitrogen and stored at −80°C until use. In this study, two miRNA libraries were constructed. Total RNAs were extracted from the backfat of three fetal and three adult Chinese Qinchuan bovines, which were pooled respectively. Subsequently, low molecular weight RNAs were separated by 15% polyacrylamide gel electrophoresis (PAGE), and RNA molecules in the range of 18–30 nt were enriched and ligated with proprietary adapters to the 5′ and 3′ termini. A reverse transcription reaction followed by low-cycle PCR was performed to obtain sufficient product for Solexa technology (Beijing Genomics Institute, China).

### Small RNA sequence analysis

After clearing away the 3′ adaptor sequence and removing redundancies and reads smaller than 18 nt, the clean reads were screened against and mapped to the latest bovine genome assembly [http://hgdownload.cse.ucsc.edu/goldenPath/bosTau6/bigZips/bosTau6.fa.gz] using the program SOAP [23]. To identify sequences originating from protein-coding genes, repeats, rRNA, tRNA, snRNA, and snoRNA, we used bovine mRNA [http://hgdownload.cse.ucsc.edu/goldenPath/bosTau6/database/refGene.txt.gz] and CDS [http://hgdownload.cse.ucsc.edu/goldenPath/bosTau6/bigZips/refMrna.fa.gz], RepeatMasker [http://www.repeatmasker.org] and Sanger Rfam data (version 10.1). Subsequently, the remaining reads were searched against the Sanger miRBase (version 19.0) to identify the conserved miRNAs. Only those small RNAs whose mature and precursor sequences perfectly matched known bovine miRNAs in the miRBase were considered conserved miRNAs. To discover potential novel miRNA precursor sequences, unique sequences that have more than 10 hits to the genome or that match to known non-coding RNAs were removed. Then, the flanking sequences (150 nt upstream and downstream) of each unique sequence were extracted for secondary structure analysis with Mfold [http://www.bioinfo.rpi.edu/applications/mfold] and evaluated by Mireap [http://sourceforge.net/projects/mireap/]. Specifically, the miRNA candidates that passed Mireap were deemed as highly probable if their corresponding miRNA*s were also found in the small RNA libraries. After prediction, the resulting potential miRNA loci were examined carefully based on the distribution and numbers of small RNAs on the entire precursor regions. Those sequences residing in the stem region of the stem-loop structure and ranging between 20–22 nt with free energy hybridization lower than −20 kcal/mol were considered [65].

### MicroRNA expression analysis

A comparison of the known miRNA expression in the two samples was conducted to determine the differentially expressed miRNAs. The samples' miRNA expression was shown by plotting the log2-ratio figure and scatter plots. The procedures are shown below: (1) Normalize the expression of miRNA in two samples (fetal and adult backfat) to get the expression of transcripts per million. Normalized expression (NE)  =  Actual miRNA count/Total count of clean reads. (2) Calculate the fold-changes and P-values from the normalized expression. Then, generate the log2-ratio and scatter plots. Fold-change formula: Fold_change  =  log2 (fetal NE/adult NE). P-value formula: 
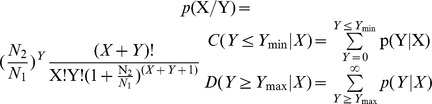


The x and y represent normalized expression levels, and N1 and N2 represent the total count of clean reads of a given miRNA in small RNA libraries of the fetal and adult stages, respectively. Stem-loop real-time reverse transcription polymerase chain reaction (RT-PCR) with SYBR Green was used for the analysis of miRNA expression [66]. Total RNA (1 µg from tested tissues) was converted to cDNA with a RT primer mixture (250 nM) using a PrimeScript® RT reagent Kit with gDNA Eraser (TaKaRa, Dalian, China). The cDNA was then used for the real-time PCR quantification of miRNA using the miRNA-specific primer and the universal primer. The bovine ribosomal protein S18 (RPS18) (GenBank NO. NM_001033614.1) gene was used as an endogenous control. The primers for miRNAs and the control gene are listed in Table S12. Real-time quantitative PCR was performed using a Bio-Rad CFX 96™ Real Time Detection System and SYBR Green PCR Master Mix (TaKaRa, Dalian, China) in a 20 µl reaction. All reactions were carried out in triplicate. The threshold cycle (Ct) was defined as the cycle number at which the fluorescence intensity passed a predetermined threshold. The quantification of each miRNA relative to the RPS18 gene was calculated using the equation: N = 2^−ΔΔCt^.

### Target gene prediction

A search for miRNA target genes was performed using an approach described earlier [67], [68], and the rules used for target prediction include the following six aspects: (1) no more than four mismatches between the sRNA & target (G-U bases count as 0.5 mismatches); (2) no more than two adjacent mismatches in the miRNA/target duplex; (3) no adjacent mismatches in positions 2∼12 of the miRNA/target duplex (5′ of miRNA); (4) no mismatches in positions 10∼11 of the miRNA/target duplex; (5) no more than 2.5 mismatches in positions 1∼12 of the miRNA/target duplex (5′ of miRNA); (6) the minimum free energy (MFE) of the miRNA/target duplex should be greater than or equal to 75% of the MFE of the miRNA bound to its perfect.

### Availability of supporting data

The data set supporting the results of this article is available in the NCBI Gene Expression Omnibus (GEO, http://www.ncbi.nlm.nih.gov/geo/) repository, with access number GSE48569.

## Supporting Information

Figure S1 Nucleotide bias of sRNA tags. *Note*: miRNA nucleotide bias of fetal bovine backfat (A) and adult bovine backfat (B), respectively.
(DOC)

Figure S2 The differentital expressions of bovine conserved (A) and novel (B) miRNAs between adult bovine muscle and adult bovine backfat tissues were shown. Note: Expression level (AM): Expression level of adult bovine muscle; Expression level (AF): Expression level of adult bovine backfat. Each point in the figure represents a miRNA. Red points represent miRNAs with fold change>2, blue points represent miRNAs with 1/2< fold change≤2, green points represent miRNAs with fold change≤1/2.
(DOC)

Figure S3 The expression of miRNAs in bovine tissues and organs were detected by RT-qPCR.
(DOC)

Figure S4 GO term distribution. Standard configuration of the Blast2GO web application (http://www.blast2go.de) was applied to generate level 2 graphs for GO-term distributions to the biological process.
(DOC)

Table S1 Bovine conserved miRNAs.
(XLS)

Table S2 Summary of base edit in fetal bovine library.
(XLS)

Table S3 Summary of base edit in adult bovine library.
(XLS)

Table S4 End variants of the fetal bovine library identified in this study.
(XLS)

Table S5 Family analysis of known miRNA.
(XLS)

Table S6 The known miRNAs expression profiles between two libraries.
(XLS)

Table S7 Novel miRNAs identified in this study.
(XLS)

Table S8 The known miRNAs expression profiles between adult muscle and adult backfat libraries.
(XLS)

Table S9 Predicted targets for novel miRNAn25.
(XLS)

Table S10 Predicted targets for novel miRNAn26.
(XLS)

Table S11 The known miRNAs expression profiles between two libraries.
(XLS)

Table S12 Stem-loop RT-PCR Primers.
(XLS)

## References

[1] Lee RC, Feinbaum RL, Ambros V (1993) The *C. elegans* heterochronic gene lin-4 encodes small RNAs with antisense complementarity to lin-14. Cell 75: 843–854. 8252621 10.1016/0092-8674(93)90529-y

[2] Bartel DP (2009) MicroRNAs: Target recognition and regulatory functions. Cell 136(2): 215–233. 19167326 10.1016/j.cell.2009.01.002 PMC3794896

[3] Bartel DP (2004) MicroRNAs: genomics, biogenesis, mechanism, and function. Cell 116: 281–297. 14744438 10.1016/s0092-8674(04)00045-5

[4] Xu P, Vernooy SY, Guo M, Hay BA (2003) The Drosophila microRNA Mir-14 suppresses cell death and is required for normal fat metabolism. Current Biology 13: 790–795. 12725740 10.1016/s0960-9822(03)00250-1

[5] Esau C, Kang X, Peralta E, Hanson E, Marcusson EG, et al (2004) MicroRNA-143 regulates adipocyte differentiation. J Biol Chem 279: 52361–52365. 15504739 10.1074/jbc.C400438200

[6] Kennell JA, Gerin I, MacDougald OA, Cadigan KM (2008) The microRNA miR-8 is a conserved negative regulator of Wnt signaling. Proc Natl Acad Sci U.S.A. 105: 15417–15422. 10.1073/pnas.0807763105 PMC2563117 18824696

[7] Hyun S, Lee JH, Jin H, Nam JW, Namkoong B, et al (2009) Conserved MicroRNA miR-8/miR-200 and Its Target USH/FOG2 Control Growth by Regulating PI3K. Cell 139(6): 1096–1108. 20005803 10.1016/j.cell.2009.11.020

[8] Esau C, Davis S, Murray SF, Yu XX, Pandey SK, et al (2006) miR-122 regulation of lipid metabolism revealed by *in vivo* antisense targeting. Cell Metabolism 3: 87–98. 16459310 10.1016/j.cmet.2006.01.005

[9] Iliopoulos D, Drosatos K, Hiyama Y, Goldberg IJ, Zannis VI (2010) MicroRNA-370 controls the expression of microRNA-122 and Cpt1alpha and affects lipid metabolism. J Lipid Res. 51: 1513–1523. 10.1194/jlr.M004812 PMC3035515 20124555

[10] Gerin I, Bommer GT, McCoin CS, Sousa KM, Krishnan V, et al (2010) Roles for miRNA-378/378* in adipocyte gene expression and lipogenesis. Am J Physiol Endocrinol Metab 299: E198–E206. 20484008 10.1152/ajpendo.00179.2010 PMC2928515

[11] Lin Q, Gao Z, Alarcon RM, Ye J, Yun Z (2009) A role of miR-27 in the regulation of adipogenesis. FEBS J. 276: 2348–2358. 10.1111/j.1742-4658.2009.06967.x PMC5330386 19348006

[12] Nakanishi N, Nakagawa Y, Tokushige N, Aokic N, Matsuzakaa T, et al (2009) The up-regulation of microRNA-335 is associated with lipid metabolism in liver and white adipose tissue of genetically obese mice. Biochemical and Biophysical Research Communications 385: 492–496. 19460359 10.1016/j.bbrc.2009.05.058

[13] Najafi-Shoushtari SH, Kristo F, Li Y, Shioda T, Cohen DE, et al (2010) MicroRNA-33 and the SREBP host genes cooperate to control cholesterol homeostasis. Science 328: 1566–1569. 20466882 10.1126/science.1189123 PMC3840500

[14] Rayner KJ, Suarez Y, Davalos A, Parathath S, Fitzgerald ML, et al (2010) MiR-33 contributes to the regulation of cholesterol homeostasis. Science 328: 1570–1573. 20466885 10.1126/science.1189862 PMC3114628

[15] Gibs R, Weinstock G, Kappes S, Schook L, Skow L, et al. (2006) Bovine Genomic Sequencing Initiative.: 1–12. www.genome.gov/Pages/Research/Sequencing/SeqProposals/BovineSEQ.pdf.

[16] Powell WE, Huffman DL (1973) Predicting Chemical Composition of Beef Carcasses from Easily Obtainable Carcass Variables. J Anim Sci 36(6): 1069–1076.

[17] Zhiliang G, Satyanaryana E, Honglin J (2007) Identification and characterization of microRNAs from the bovine adipose tissue and mammary gland. FEBS Lett 581: 981–988. 17306260 10.1016/j.febslet.2007.01.081

[18] Tripurani SK, Xiao C, Salem M, Yao J (2010) Cloning and analysis of fetal ovary microRNAs in cattle. Anim Reprod Sci 120: 16–22. 20347535 10.1016/j.anireprosci.2010.03.001

[19] Tripurani SK, Lee KB, Wee G, Smith GW, Yao J (2011) MicroRNA-196a regulates bovine newborn ovary homeobox gene (NOBOX) expression during early embryogenesis. BMC Dev Biol 11: 25. 21548929 10.1186/1471-213X-11-25 PMC3103443

[20] Lingenfelter BM, Tripurani SK, Tejomurtula J, Smith GW, Yao J (2011) Molecular cloning and expression of bovine nucleoplasmin 2 (NPM2): a maternal effect gene regulated by miR-181a. Reprod Biol Endocrino l 9: 40. 10.1186/1477-7827-9-40 PMC3072940 21447182

[21] Sun J, Li M, Li Z, Xue J, Lan X, et al (2013) Identification and profiling of conservedand novel microRNAs from Chinese Qinchuan bovine longissimus thoracis. BMC Genomics 14: 42. 23332031 10.1186/1471-2164-14-42 PMC3563516

[22] Jin W, Dodson MV, Moore SS, Basarab JA, Guan LL (2010) Characterization of microRNA expression in bovine adipose tissues: a potential regulatory mechanism of subcutaneous adipose tissue development. BMC Mol Biol 11: 29. 20423511 10.1186/1471-2199-11-29 PMC2874793

[23] Li R, Li Y, Kristiansen K, Wang J (2008) SOAP: short oligonucleotide alignment program. Bioinformatics 24: 713–714. 18227114 10.1093/bioinformatics/btn025

[24] Brennecke J, Stark A, Russell RB, Cohen SM (2005) Principles of microRNA-target recognition. PLoS Biol 3: e85. 15723116 10.1371/journal.pbio.0030085 PMC1043860

[25] Xie FL, Huang SQ, Guo K, Xiang AL, Zhu YY, et al (2008) Computational identification of novel microRNAs and targets in Brassica napus. FEBS Lett 581: 1464–1474. 10.1016/j.febslet.2007.02.074 17367786

[26] Wang XW (2009) A PCR-based platform for microRNA expression profiling studies. RNA 15: 716–723. 19218553 10.1261/rna.1460509 PMC2661836

[27] Jin W, Grant JR, Stothard P, Moore SS, Guan LL (2009) Characterization of bovine miRNAs by sequencing and bioinformatics analysis. BMC Mol Bio 10: 90. 19758457 10.1186/1471-2199-10-90 PMC2761914

[28] Borchert GM, Gilmore BL, Spengler RM, Xing Y, Lanier W, et al (2009) Adenosine deamination in human transcripts generates novel microRNA binding sites. Hum. Mol. Genet 18: 4801–4807. 10.1093/hmg/ddp443 PMC2778373 19776031

[29] Rosen ED, Spiegelman BM (2006) Adipocytes as regulators of energy balance and glucose homeostasis. Nature 444: 847–853. 17167472 10.1038/nature05483 PMC3212857

[30] Yang X, Enerbäck S, Smith U (2003) Reduced expression of FOXC2 and brown adipogenic genes in human subjects with insulin resistance. Obes Res 11: 1182–1191. 14569043 10.1038/oby.2003.163

[31] Almind K, Manieri M, Sivitz WI, Cinti S, Kahn CR (2007) Ectopic brown adipose tissue in muscle provides a mechanism for differences in risk of metabolic syndrome in mice. Proc Natl Acad Sci U S A 104: 2366–2271. 17283342 10.1073/pnas.0610416104 PMC1892979

[32] Baek D, Villen J, Shin C, Camargo FD, Gygi SP, et al (2008) The impact of microRNAs on protein output. Nature 455: 64–71. 18668037 10.1038/nature07242 PMC2745094

[33] Lewis BP, Burge CB, Bartel DP (2005) Conserved seed pairing, often flanked by adenosines, indicates that thousands of human genes are microRNA targets. Cell 120: 15–20. 15652477 10.1016/j.cell.2004.12.035

[34] Teleman AA, Maitra S, Cohen SM (2006) Drosophila lacking microRNA miR-278 are defective in energy homeostasis. Genes Dev 20: 417–422. 16481470 10.1101/gad.374406 PMC1369043

[35] Lau NC, Lim LP, Weinstein EG, Bartel DP (2009) An abundant class of tiny RNAs with probable regulatory roles in Caenorhabditis elegans. Science 294: 858–862. 10.1126/science.1065062 11679671

[36] Chen Chen, Bing Deng, Mu Qiao, Rong Zheng, Jin Chai, et al (2012) Solexa Sequencing Identification of Conserved and Novel microRNAs in Backfat of Large White and Chinese Meishan Pigs. PLoS ONE 7(2): e31426. 22355364 10.1371/journal.pone.0031426 PMC3280305

[37] Shifang Z, Fuping Z, Caihong W, Xihui S, Hangxing R, et al (2013) Identification and Characterization of the miRNA Transcriptome of Ovis aries. PLoS ONE 8(3): e58905. 23516575 10.1371/journal.pone.0058905 PMC3596360

[38] Xuechun Y, Lei D, Yunchao L, Xiaofeng Z, Yang L, et al (2012) Identification and Profiling of MicroRNAs from Skeletal Muscle of the Common Carp. PLoS ONE 7(1): e30925. 22303472 10.1371/journal.pone.0030925 PMC3267759

[39] Zhibin J, Guizhi W, Zhijing X, Jianmin W, Chunlan Z, et al (2012) Identification of Novel and Differentially Expressed MicroRNAs of Dairy Goat Mammary Gland Tissues Using Solexa Sequencing and Bioinformatics. PLoS ONE 7(11): e49463. 23166677 10.1371/journal.pone.0049463 PMC3498112

[40] Jinming H, Zhihua J, Qiuling L, Qinlei H, Changfa W, et al (2011) Solexa Sequencing of Novel and Differentially Expressed MicroRNAs in Testicular and Ovarian Tissues in Holstein Cattle. Int. J. Biol. Sci. 7: 1016–1026. 10.7150/ijbs.7.1016 PMC3164151 21912509

[41] Swamy K, Tripurani, Caide X, Mohamed Salem, Jianbo Y (2010) Cloning and analysis of fetal ovary microRNAs in cattle. Animal Reproduction Science 120: 16–22. 20347535 10.1016/j.anireprosci.2010.03.001

[42] Wang L, Liu H, Li D, Chen H (2011) Identification and characterization of maize microRNAs involved in the very early stage of seed germination. BMC Genomics 12: 154. 21414237 10.1186/1471-2164-12-154 PMC3066126

[43] Kawahara Y, Zinshteyn B, Sethupathy P, Iizasa H, Hatzigeorgiou AG, et al (2007) Redirection of silencing targets by adenosine-to-inosine editing of miRNAs. Science 315: 1137–1140. 17322061 10.1126/science.1138050 PMC2953418

[44] Landgraf P, Rusu M, Sheridan R (2007) A mammalian microRNA expression atlas based on small RNA library sequencing. Cell 129: 1401–1414. 17604727 10.1016/j.cell.2007.04.040 PMC2681231

[45] Ramachandran V, Chen X (2008) Degradation of microRNAs by a family of exoribonucleases in Arabidopsis. Science 321: 1490–1492. 18787168 10.1126/science.1163728 PMC2570778

[46] Guo X, Su B, Zhou Z, Sha J (2009) Rapid evolution of mammalian X-linked testis microRNAs. BMC Genomics 10: 97. 19257908 10.1186/1471-2164-10-97 PMC2660371

[47] Buchold GM, Coarfa C, Kim J, Milosavljevic A, Gunaratne PH, et al (2010) Analysis of microRNA expression in the prepubertal testis. PLoS One 5: e15317. 21206922 10.1371/journal.pone.0015317 PMC3012074

[48] Girard M, Jacquemin E, Munnich A, Lyonnet S, Henrion CA (2008) MiR-122, a paradigm for the role of microRNAs in the liver. J Hepatol 48: 648–656. 18291553 10.1016/j.jhep.2008.01.019

[49] Trajkovski M, Hausser J, Soutschek J, Bhat B, Akin A, et al (2011) MicroRNAs 103 and 107 regulate insulin sensitivity. Nature 474: 649–653. 21654750 10.1038/nature10112

[50] Juling J, Jinsheng Z, Guangcun H, Jin Q, Xueqing W, et al (2009) Over expressed microRNA-27a and 27b influence fat accumulation and cell proliferation during rat hepatic stellate cell activation. FEBS Letters 583: 759–766. 19185571 10.1016/j.febslet.2009.01.034

[51] Sang YK, A Young Kim, Hyun WL, You HS, Gha YL, et al (2010) miR-27a is a negative regulator of adipocyte differentiation via suppressing PPARγ expression. Biochemical and Biophysical Research Communications 392: 323–328. 20060380 10.1016/j.bbrc.2010.01.012

[52] Tingwan S, Mingui F, Angie LB, Steven AK, David JM (2009) MicroRNA let-7 Regulates 3T3-L1 Adipogenesis. Mol Endocrinol 23(6): 925–931. 19324969 10.1210/me.2008-0298 PMC2691679

[53] Buck AH, Perot J, Chisholm MA, Kumar DS, Tuddenham L, et al (2010) Post-transcriptional regulation of miR-27 in murine cytomegalovirus infection. RNA 16: 307–315. 20047990 10.1261/rna.1819210 PMC2811660

[54] Moncini S, Salvi A, Zuccotti P, Viero G, Quattrone A, et al (2011) The Role of miR-103 and miR-107 in Regulation of CDK5R1 Expression and in Cellular Migration. PLoS ONE 6(5): e20038. 21625387 10.1371/journal.pone.0020038 PMC3100319

[55] Reinhart BJ, Slack FJ, Basson M, Pasquinelli AE, Bettinger JC, et al (2000) The 21-nucleotide let-7 RNA regulates developmental timing in Caenorhabditis elegans. Nature 403: 901–906. 10706289 10.1038/35002607

[56] Gao Y, Schug J, McKenna LB, Le LJ, Kaestner KH, et al (2011) Tissuespecific regulation of mouse MicroRNA genes in endoderm-derived tissues. Nucleic Acids Research 39: 454–463. 20843784 10.1093/nar/gkq782 PMC3025567

[57] Lagos QM, Rauhut R, Yalcin A, Meyer J, Lendeckel W, et al (2002) Identification of Tissue-Specific MicroRNAs from Mouse. Current Biology 12: 735–739. 12007417 10.1016/s0960-9822(02)00809-6

[58] Liang Y, Ridzon D, Wong L, Chen C (2007) Characterization of microRNA expression profiles in normal human tissues. BMC Genomics 8: 166. 17565689 10.1186/1471-2164-8-166 PMC1904203

[59] Hishikawa D, Hong YH, Roh SG, Miyahara H, Nishimura Y, et al (2005) Identification of genes expressed differentially in subcutaneous and visceral fat of cattle, pig, and mouse. Physiological Genomics 21: 343–350. 15784696 10.1152/physiolgenomics.00184.2004

[60] Creighton CJ, Benham AL, Zhu H, Khan MF, Reid JG, et al (2010) Discovery of novel microRNAs in female reproductive tract using next generation sequencing. PLoS One 5: e9637. 20224791 10.1371/journal.pone.0009637 PMC2835764

[61] Choi JH, Banks AS, Estall JL, Kajimura S, Bostrom P, et al (2010) Obesity-linked phosphorylation of PPARγ by cdk5 is a direct target of the anti-diabetic PPARγ ligands. Nature 466: 451–456. 20651683 10.1038/nature09291 PMC2987584

[62] Leone TC, Weinheimer CJ, Kelly DP (1999) A critical role for the peroxisome proliferator-activated receptor α (PPARα) in the cellular fasting response: the PPARα-null mouse as a model of fatty acid oxidation disorders. Proc Natl Acad Sci USA 96: 7473–7478. 10377439 10.1073/pnas.96.13.7473 PMC22110

[63] Schultz JR, Tu H, Luk A, Repa JJ, Medina JC, et al (2000) Role of LXRs in control of lipogenesis. Genes Dev 14: 2831–283. 11090131 10.1101/gad.850400 PMC317060

[64] Richardson C, Jones PC, Barnard V, Hebert CN, Terlecki S, et al (1990) Estimation of the developmental age of the bovine fetus and newborn calf. Vet. Rec. 126: 279–284. 2343510

[65] Ambros V, Bartel B, Bartel DP, Burge CB, Carrington JC, et al (2003) A uniform system for microRNA annotation. RNA 9(3): 277–279. 12592000 10.1261/rna.2183803 PMC1370393

[66] Chen C, Ridzon DA, Broomer AJ, Zhou Z, Lee DH, et al (2005) Real-time quantification of microRNAs by stem-loop RT-PCR. Nucleic Acids Res 33: e179. 16314309 10.1093/nar/gni178 PMC1292995

[67] Allen E, Xie ZX, Gustafson AM, Carrington JC (2005) microRNA-Directed Phasing during Trans-Acting siRNA Biogenesis in Plants. Cell 121: 207–221. 15851028 10.1016/j.cell.2005.04.004

[68] Schwab R, Palatnik JF, Riester M, Schommer C, Schmid M, et al (2005) Specific Effects of MicroRNAs on the Plant Transcriptome. Developmental Cell 8: 517–527. 15809034 10.1016/j.devcel.2005.01.018

