# Supplementary material for: Discovery of Novel and Differentially Expressed MicroRNAs between Fetal and Adult Backfat in Cattle
Source: PLoS One. 2014 Feb 28;9(2):e90244. doi: 10.1371/journal.pone.0090244 (PMC3938653; doi:10.1371/journal.pone.0090244)
Supplement: Figure S1 — Nucleotide bias of sRNA tags. Note: miRNA nucleotide bias of fetal bovine backfat (A) and adult bovine backfat (B), respectively. (DOC) [file pone.0090244.s001.doc]

**(A)**

[
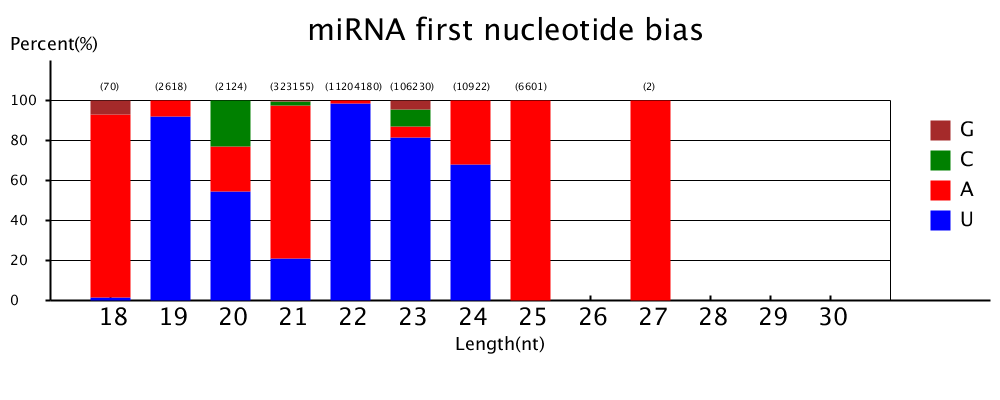
](file:///E:/第二次高通量测序/全部结果/BGI_SmallRNA_report/BGI_SmallRNA_report/Files/BGI_graph/match_hairpin/FF/hairpin_first_base.png)[
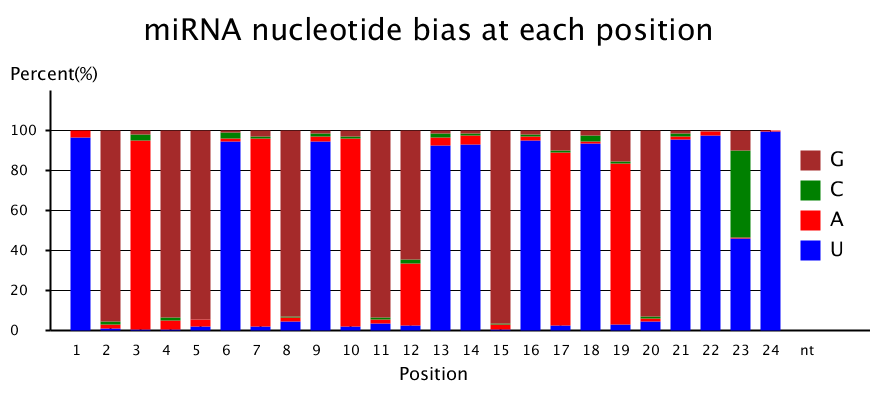
](file:///E:/第二次高通量测序/全部结果/BGI_SmallRNA_report/BGI_SmallRNA_report/Files/BGI_graph/match_hairpin/FF/hairpin_miRNA_base_bias.png)

**(B)**

[
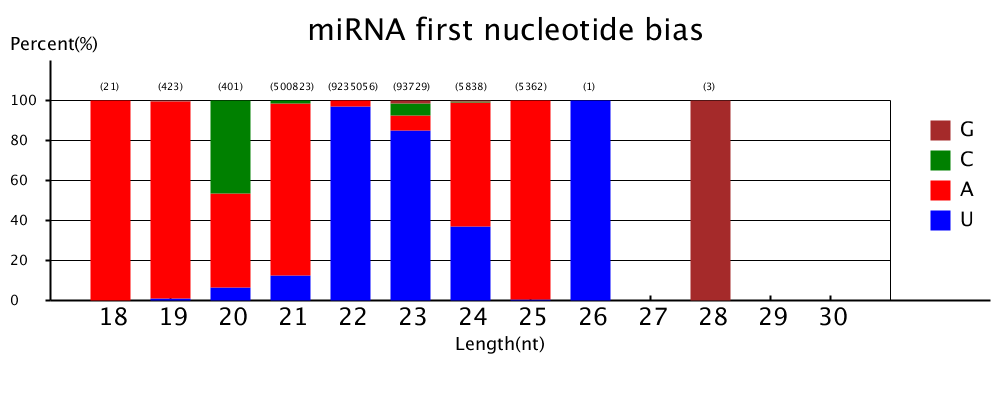
](file:///E:/第二次高通量测序/全部结果/BGI_SmallRNA_report/BGI_SmallRNA_report/Files/BGI_graph/match_hairpin/AF/hairpin_first_base.png)[
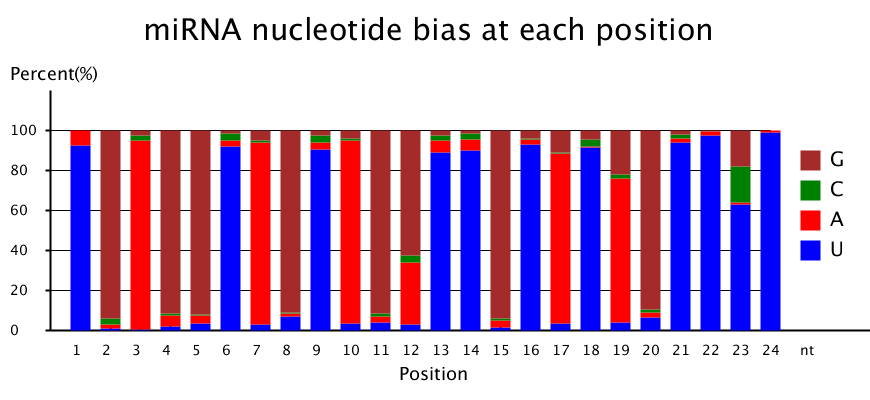
](file:///E:/第二次高通量测序/全部结果/BGI_SmallRNA_report/BGI_SmallRNA_report/Files/BGI_graph/match_hairpin/AF/hairpin_miRNA_base_bias.png)

**Figure S1** Nucleotide bias of sRNA tags. *Note:* miRNA nucleotide bias of fetal bovine backfat (A) and adult bovine backfat (B), respectively.
