# Supplementary material for: Discovery of Novel and Differentially Expressed MicroRNAs between Fetal and Adult Backfat in Cattle
Source: PLoS One. 2014 Feb 28;9(2):e90244. doi: 10.1371/journal.pone.0090244 (PMC3938653; doi:10.1371/journal.pone.0090244)
Supplement: Figure S2 — The differentital expressions of bovine conserved (A) and novel (B) miRNAs between adult bovine muscle and adult bovine backfat tissues were shown. Note: Expression level (AM): Expression level of adult bovine muscle; Expression level (AF): Expression level of adult bovine backfat. Each point in the figure represents a miRNA. Red points represent miRNAs with fold change>2, blue points represent miRNAs with 1/2< fold change≤2, green points represent miRNAs with fold change≤1/2. (DOC) [file pone.0090244.s002.doc]

1. (B)


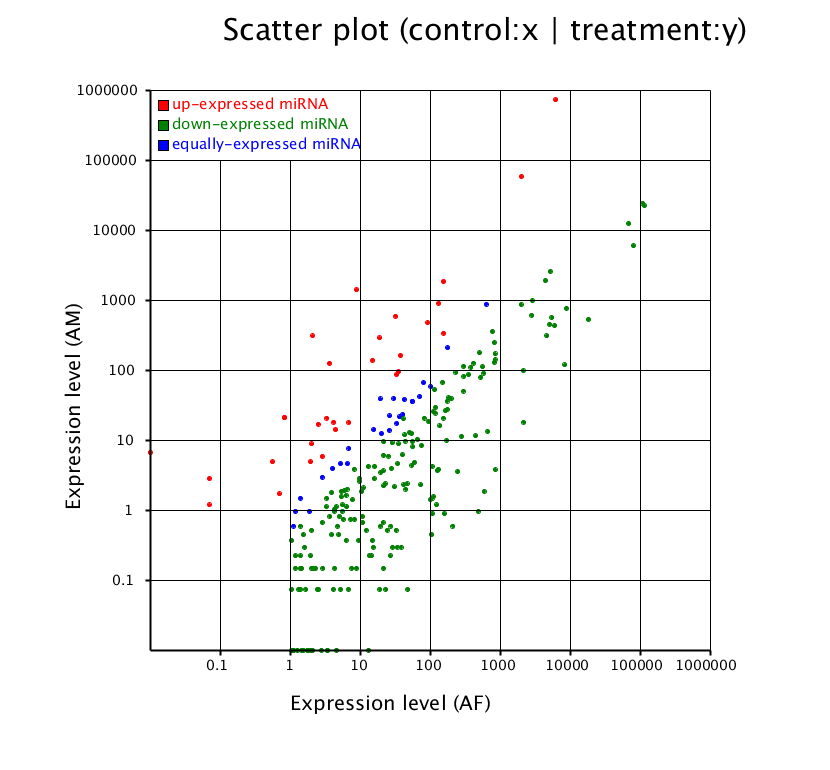

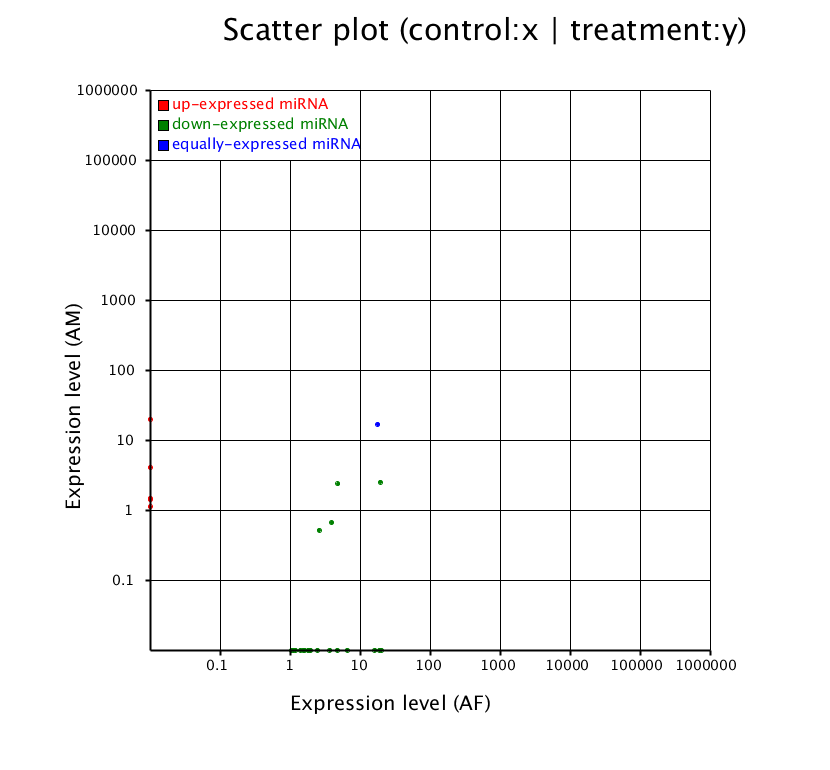


**Figure S2** The differentital expressions of bovine conserved (A) and novel (B) miRNAs between adult bovine muscle and adult bovine backfat tissues were shown. Note: Expression level (AM): Expression level of adult bovine muscle; Expression level (AF): Expression level of adult bovine backfat. Each point in the figure represents a miRNA. Red points represent miRNAs with fold change＞2, blue points represent miRNAs with 1/2＜fold change≤2, green points represent miRNAs with fold change≤1/2.
