# Supplementary material for: Discovery of Novel and Differentially Expressed MicroRNAs between Fetal and Adult Backfat in Cattle
Source: PLoS One. 2014 Feb 28;9(2):e90244. doi: 10.1371/journal.pone.0090244 (PMC3938653; doi:10.1371/journal.pone.0090244)
Supplement: Figure S3 — The expression of miRNAs in bovine tissues and organs were detected by RT-qPCR. (DOC) [file pone.0090244.s003.doc]

**Figure S3** The expression of miRNAs in bovine tissues and organs were detected by RT-qPCR.
