# Supplementary material for: Discovery of Novel and Differentially Expressed MicroRNAs between Fetal and Adult Backfat in Cattle
Source: PLoS One. 2014 Feb 28;9(2):e90244. doi: 10.1371/journal.pone.0090244 (PMC3938653; doi:10.1371/journal.pone.0090244)
Supplement: Figure S4 — GO term distribution. Standard configuration of the Blast2GO web application (http://www.blast2go.de) was applied to generate level 2 graphs for GO-term distributions to the biological process. (DOC) [file pone.0090244.s004.doc]

**Figure S4** GO term distribution. Standard configuration of the Blast2GO web application ([http://www.blast2go.de](http://www.blast2go.de/)) was applied to generate level 2 graphs for GO-term distributions to the biological process.
